# Association between early recurrences of atrial tachyarrhythmias and long-term outcomes in patients after repeat atrial fibrillation ablation

**DOI:** 10.1007/s10840-021-00987-z

**Published:** 2021-04-07

**Authors:** Douglas Darden, Omar Aldaas, Chaitanya L. Malladi, Praneet S. Mylavarapu, Muhammad Bilal Munir, Frederick T. Han, Kurt S. Hoffmayer, Farshad Raissi, Gordon Ho, David Krummen, Gregory K. Feld, Jonathan C. Hsu

**Affiliations:** Division of Cardiology, Department of Medicine, University of California, San Diego, 9452 Medical Center Drive, 3rd Floor, Room 3E-417, La Jolla, CA 92037 USA

**Keywords:** Early recurrence, Blanking period, Repeat ablation, Pulmonary vein isolation, Atrial fibrillation

## Abstract

**Purpose:**

Early recurrence of atrial tachyarrhythmia (ER) is predictive of late recurrence of atrial tachyarrhythmia (LR) after first-time atrial fibrillation (AF) ablation, but the association in patients undergoing repeat AF ablation is unknown. We aim to determine the incidence and prognostic significance of ER after repeat ablation.

**Methods:**

A total of 259 consecutive patients (mean age 64 years, 75.3% male) undergoing repeat AF ablation with complete follow-up data were included at a single institution from 2010 to 2015. ER and LR were defined as atrial tachyarrhythmia (AF, atrial flutter or atrial tachycardia) > 30 s within the 3-month blanking period (BP) and after the 3-month BP, respectively.

**Results:**

ER occurred in 79/259 (30.5%), and LR occurred in 138/259 (53%) at a median follow-up of 1221 (IQR: 523–1712) days. Four-year freedom from LR was 22% and 56% in patients with and without ER, respectively (*p* < 0.001). After multivariate adjustment, ER was strongly associated with LR, cardioversion post BP, and repeat ablation, but not associated with hospitalization. Compared to those with no ER, there was a higher risk of LR when ER occurred within the first month of the BP [month 1: hazard ratio (HR) 2.32, confidence interval (CI) 1.57–3.74, *p* < 0.001; month 2: HR 2.01, CI 1.13–3.83, *p* = 0.02; month 3: HR 1.46, CI 0.5–3.36, *p* = 0.37], however the prediction of LR based on timing within the BP was poor (area under curve 0.64).

**Conclusion:**

Following repeat AF ablation, ER is strongly associated with LR, cardioversion post BP, and repeat ablation.

## Introduction

Catheter ablation for drug-refractory symptomatic atrial fibrillation (AF) is an effective treatment; however, early recurrence of atrial tachyarrhythmias (ER) after successful pulmonary vein isolation (PVI) is common [1]. Although ER may occur in over a third of patients, it does not necessarily imply ablation failure [2]. Since not all patients with ER experience late recurrence of atrial tachyarrhythmias (LR), it is thought that ER may result from transient effects of post-ablation inflammation and autonomic imbalance [3]. Therefore, consensus guidelines have proposed a 3-month post procedural blanking period (BP) in which recurrences should not prompt reintervention [1].

However, several studies involving patients following *de novo* PVI have reported a strong association between ER and LR [4–6]. Furthermore, the timing of ER within the BP has important predictive implications. Specifically, those with ER that occurs later in the BP are at a significantly higher risk of LR, advocating consideration of shortening of the BP duration [7–9].

Those with LR frequently pursue repeat ablation and clinicians subsequently repeatedly apply the recommended BP to this cohort. Although repeat ablation increases arrhythmia-free survival considerably, no previous study has examined the significance of ER in patients undergoing repeat ablation [10]. The aim of the present study is to determine the incidence, clinical predictors, and prognostic significance of ER and the timing of ER in patients undergoing repeat AF ablation.

## Methods

### Study population

Consecutive patients undergoing repeat radiofrequency ablation for repeat atrial fibrillation with complete follow-up data at the University of California, San Diego (UCSD), Health System between October 2009 and March 2015 were analyzed from the UCSD AF Ablation Registry, an observational, retrospective cohort study. The UCSD AF Ablation Registry was designed as a clinical registry of all patients undergoing left atrial ablation procedures for atrial arrhythmias at UCSD, a single academic center, as captured by a procedural database (Perminova, Inc., San Diego, CA) to collect patient, provider, and intra-procedural characteristics. All AF ablation procedures captured by the registry were linked to clinical encounters as recorded by the electronic medical record at the institution (Epic, Verona, WI). Data on baseline demographics, medical history, laboratory data, medications, and cardiovascular implantable devices were collected. Intra-procedural registry reports were reviewed to determine fluoroscopy and procedure times and ablation lesion sets.

All patients undergoing repeat atrial fibrillation ablation had a history of symptomatic, medically refractory, paroxysmal or persistent AF after a first PVI ablation procedure. Patients with atypical atrial flutter and atrial tachycardia as the primary indication and those with > 2 previous AF catheter ablations were excluded. The study was approved by the UCSD Institutional Review Board.

### Procedural details

Informed consent was obtained prior to all ablation procedures. General anesthesia was used in all cases. Intravenous heparin was used to target an activated clotting time of 350–400 s. A transseptal puncture was performed under direct visualization with intracardiac echocardiography guidance. Electroanatomic mapping systems were used in all cases (CARTO™ (Biosense-Webster Inc, Diamond Bar, CA; or Ensite™, St Jude Medical, Inc., Minneapolis, MN). Esophageal position and temperature were monitored during all left atrial ablations using a multipolar temperature probe (Circa S-Cath, Circa Scientific, Inc., Englewood, CO) positioned in the esophagus behind the left atrium at the level of the ablation catheter, in order to avoid any temperature rise above 38 °C. If recovery of conduction from PVs was observed, repeat PVI was performed using a segmental, circumferential, or both approaches. Additional lesion sets outside the previously ablated regions were performed at the discretion of the operator, including left atrial roof line, mitral valve isthmus line, coronary sinus ablation, and complex fractional atrial electrogram ablation. Additionally, organized atrial arrhythmias such as atrial flutter (AFL) or atrial tachycardia (AT) were mapped and ablated, as were ectopic premature atrial contractions. Closed and open irrigated and non-contact and contact force sensing catheters were also used at the discretion of the operator. The endpoint of PVI was elimination of all PV potentials and demonstration entrance and exit block by pacing after a 30-min waiting period and elimination of a trigger or a line of bidirectional conduction block if adjunctive ablations were performed.

### Post-ablation follow-up

After ablation, patients were hospitalized overnight and usually discharged the day after the procedure if stable. Oral anticoagulation was restarted in all patients after ablation. Most patients were discharged on the antiarrhythmic drug regimen they had been on before ablation and were most commonly maintained on this medication for up to 90 days, after which it was discontinued per provider discretion.

After discharge, all patients were seen in the UCSD outpatient arrhythmia clinic typically at 1, 6, 12, and 24 months after ablation and at 12-month intervals thereafter. A clinical assessment and a 12-lead electrocardiogram were routinely performed at each visit. Patients had routine ambulatory ECG monitoring (24-h Holter monitor, extended ambulatory electrocardiogram monitoring, or event monitoring) at 6, 12, and 24 months after ablation. Additional monitoring was provided in any patient reporting symptoms suggesting recurrent arrhythmia.

### Outcomes

Documentation of any arrhythmic episode was based on ECG, Holter or event monitor data, or implantable device recordings. ER was defined as any atrial tachyarrhythmia (AF, AFL, AT) lasting > 30 s within the first 3 months after the repeat ablation procedure. LR was defined as recurrence of any atrial tachyarrhythmia (AF, AFL, AT) > 30 s occurring > 3 months after repeat procedure.

Additional clinical outcomes were determined during follow-up and included in-hospital adverse events, direct current cardioversion (DCCV), repeat ablation, and all-cause hospitalizations. Adverse events were recorded in the registry and included access site complications (i.e., bleeding, groin hematoma, pseudoaneurysm, and arterio-venous fistula), cardiac perforation or tamponade, stroke or transient ischemic attack, pericarditis, myocardial infarction, atrio-esophageal fistula, phrenic nerve paralysis, and pulmonary vein stenosis.

### Statistical analysis

The baseline characteristics of those with ER versus no ER were reported as means ± one standard deviation and frequency (*n* and %) for continuous and categorical variables, respectively. Chi-square and *t-*tests were used for the between-group comparisons of the categorical and continuous variables, respectively. Participant characteristics that differed between those with and without ER in univariate analysis (at a *p* level < 0.20) were entered into a Cox regression model with backward selection method to identify clinical predictors of ER. A significance level of *p* ≤ 0.05 was used for variable removal during the selection process.

Time to recurrence and event-free survival curves were analyzed using the Kaplan-Meier method with a 3-month BP and log-rank significance testing. Cox proportional hazards modeling was used to analyze arrhythmia-free survival with a 3-month BP with results presented as hazard ratios (HR) with 95% confidence intervals (CI), after verifying proportionality assumptions. Patients who were lost to follow-up were censored at the date of last known follow-up. Variables in the multivariable model were chosen a priori based on the potential for confounding from prior literature and availability within the dataset, including age, sex, body mass index, hypertension, diabetes, obstructive sleep apnea, chronic kidney disease, heart failure, type of atrial fibrillation (paroxysmal vs persistent), roof line ablation, mitral isthmus line ablation, and cavotricuspid isthmus ablation. Finally, receiver operator characteristic (ROC) curve analysis was performed to assess the correlation between the timing of ER and LR. Missing values were minimal (except in the case of the echocardiographic parameters) and roughly equivalent between groups for all variables and were thus omitted. All analyses were performed using IBM SPSS Statistics Version 26 (IBM Corp, NY, USA).

## Results

### Differences in clinical characteristics in patients with and without early recurrence of atrial tachyarrhythmias

The baseline characteristics of 259 patients (mean age 63.6 years, 75.3% male) undergoing repeat PVI are presented in Table 1. A total of 180 (69.5%) patients had no ER, while 79 (30.5%) patients had ER. There were no significant differences in age, total duration of AF, paroxysmal AF, co-morbidities, or echocardiographic parameters. Patients with ER were more likely to be prescribed an antiarrhythmic drug (AAD) in the BP (initiated or continued at the time of index ablation hospitalization) than those without ER (89.9% vs 75.4%, respectively, *p* = 0.008). No significant differences were observed between type of AAD prescription or other cardiac medications.

**Table 1:** Baseline characteristics of patients with and without recurrence of early atrial tachyarrhythmias (ER) during the 3-month blanking period

Variable	ER (*N* = 79)	No ER (*N* = 180)	*p* value
Age, y	64.3 (± 9.6)	63.2 (± 10.7)	0.47
Male	64 (81)	131 (72.8)	0.16
Body mass index (kg/min)	29.4 (± 5.6)	28.0 (± 5.4)	0.056
Total duration of AF, days	2330.4 (± 1839.3)	2284.7 (± 1818.5)	0.86
Paroxysmal AF	49 (62.0)	133 (73.9)	0.054
AT/AFL	6 (7.6)	15 (8.3)	0.87
CHA_2_DSVA_2_SC score	2.1 (± 1.5)	1.9 (± 1.5)	0.32
Permanent pacemaker	6 (7.6)	19 (10.6)	0.46
Cardiac resynchronization therapy	3 (3.8)	8 (4.4)	0.81
Comorbidities			
Hypertension	44 (55.7)	96 (3.6)	0.76
Hyperlipidemia	32 (40.5)	63 (35.2)	0.42
Diabetes	8 (10.1)	17 (9.4)	0.86
Heart failure	14 (17.7)	21 (11.7)	0.19
Chronic kidney disease	1 (1.3)	3 (1.7)	0.80
Chronic obstructive lung disease	0 (0)	6 (4.4)	0.10
Obstructive sleep apnea	9 (11.4)	25 (13.9)	0.58
Prior stroke	11 (13.9)	15 (8.3)	0.17
Coronary artery disease	13 (16.5)	34 (18.9)	0.64
Prior percutaneous coronary intervention	7 (8.9)	20 (11.0)	0.59
Prior coronary artery bypass graft surgery	1 (1.3)	6 (3.3)	0.35
Smoker	13 (16.5)	27 (15.0)	0.77
Medications			
Antiarrhythmic drug during blanking period	71 (89.9)	135 (75.4)	0.008
Flecainide	0 (0)	1 (0.6)	0.10
Propafenone	5 (6.3)	10 (5.6)	0.81
Sotalol	20 (25.3)	47 (26.1)	0.89
Dronedarone	9 (11.4)	11 (6.1)	0.14
Amiodarone	10 (12.7)	17 (9.4)	0.44
Dofetilide	4 (5.1)	8 (4.4)	0.83
Beta blocker	39 (49.4)	81 (45.0)	0.52
Calcium channel blocker	18 (22.8)	54 (30.0)	0.23
Digoxin	9 (11.4)	12 (6.7)	0.20
ACEi/ARB	25(31.6)	35.5 (35.6)	0.80
Echocardiographic parameters	*N* = 32	*N* = 75	
Left atrial diameter, mm	4.1 (± 0.8)	4.3 (± 0.6)	0.32
Left atrial volume index	32.8 (± 15.2)	33.0 (7.6)	0.95
Left ventricular ejection fraction	59.1 (± 10.4)	61.5 (± 8.0)	0.38
Left ventricular end diastolic diameter	4.7 (± 0.6)	4.9 (± 0.7)	0.57

Abbreviations: *ER*, early recurrence of atrial tachyarrhythmia; *AF*, atrial fibrillation; *AT*, atrial tachycardia; *AFL*, atrial flutter; *ACEi*, ACE inhibitor; *ARB*, angiotensin receptor blocker

### Procedural characteristics and complications

As shown in Table 2, procedure times were significantly longer in those with ER compared to those with no ER (258 ± 69 vs 218 ± 66 mins, respectively, *p* < 0.001), while no differences were observed with total fluoroscopy time. Pulmonary vein reconnection was present in 75% of those with and 78% of those without ER (*p* = 0.8). Those with ER were more likely to have undergone left atrial roof line ablation (46.8% vs 31.1%, *p* = 0.014) and mitral isthmus line ablation (43.0% vs 25.6%, *p* = 0.005) than those with no ER. Complication rates were low with no significant differences between those with and without ER.

**Table 2:** Procedural characteristics and complications in patients with and without subsequent recurrence of early atrial tachyarrhythmia (ER)

Variable	ER (*n* = 79)	No ER (*n* = 180)	*p* value
Procedural characteristics			
Total hospital stay (days)	1.4 (± 1.0)	1.2 (± 0.6)	0.10
Total procedure time (mins)	258 (± 69)	218 (± 66)	< 0.001
Fluoroscopy time (mins)	74.8 (± 27.2)	64.6 (± 22.8)	0.28
Pulmonary vein reconnection	59 (74.6)	140 (77.8)	0.81
Successful PVI	76 (96.2)	175 (97.2)	0.86
Ablation type			
Segmental	33 (44)	110 (63.6)	0.004
Circumferential	19 (25.3)	21 (12.1)	0.02
Both	23 (30.7)	42 (24.3)	0.35
CTI flutter line	35 (44.3)	78 (43.3)	0.83
LA roof line	37 (46.8)	56 (31.1)	0.014
Mitral isthmus line	34 (43.0)	46 (25.6)	0.005
CFAE ablation	7 (9.1)	10 (5.7)	0.33
Procedural complications			
Access site complication	9 (11.4)	10 (5.6)	0.12
Cardiac perforation/tamponade	0 (0)	0 (0)	NA
Stroke/transient ischemic attack	0 (0)	2 (1.1)	0.89
Pericarditis	1 (1.3)	1 (0.6)	0.36
Other complications*	0 (0)	0 (0)	NA

*Other complications included myocardial infarction, atrioesophageal fistula, phrenic nerve paralysis, and pulmonary vein stenosis

Abbreviations: *PVI*, pulmonary vein isolation; *CTI*, cavotricuspid isthmus; *LA*, left atrial; *CFAE*, complex fractionated atrial electrogram

### Predictors of ER and LR

In multivariable Cox regression, there were no significant predictors of ER. Significant predictors of LR after multivariable adjustment included roof line ablation (HR: 1.83, 95% CI 1.20–2.7, *p* = 0.005) and ER (HR: 2.3, 95% CI 1.62–3.36, *p* < 0.001).

### Outcomes

**Fig. 1:**
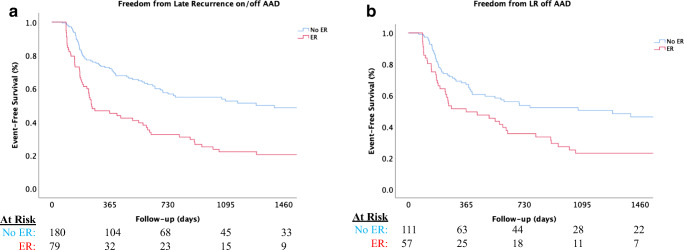
Title: Kaplan-Meier plots comparing clinical outcomes stratified by early recurrence of atrial tachyarrhythmias within the blanking period versus no early recurrence of atrial tachyarrhythmias during the blanking period. **a** Freedom from late recurrence (LR) on or off anti-arrhythmic drug (AAD); **b** Freedom from LR off AAD. Caption: Time zero indicates 91^st^ day (after blanking period)

During a median follow-up of 1221 (interquartile range [IQR]: 523–1712) days, LR on or off AAD occurred in 135/259 (52.1%). LR occurred in 60/79 (75.9%) of those with ER and 75/180 (41.7%) of those without ER, while 19/124 (15.3%) of those with ER did not experience LR. The median time to LR was 251 (IQR: 145–1014) days in those with ER compared to 1286 (IQR: 293–2468) days in those without ER. As shown in Fig. 1a, Kaplan-Meier analysis showed a higher risk of LR in those with ER with approximately 78% of those with ER and 44% of those without ER experiencing LR at 4 years (log rank *p* < 0.001). After multivariate adjustment, the risk of LR was over twofold higher in those with ER compared to those without ER (HR: 2.36, CI 1.54–3.32, *p* < 0.001). Furthermore, 168/259 (64.9%) patients underwent AAD discontinuation after the repeat ablation, including 57/79 (72.2%) of those with ER and 111/180 (61.7%) of those without ER. At 4 years of follow-up, 79% of those with ER and 46% of those without ER experienced LR at 4 years (long rank *p* = 0.003), as shown in Fig. 1b. A strong association was also observed with an increased risk of LR off AAD in those with ER (Table 3).

**Table 3:** Cox-proportional hazards models for the association of early recurrence of atrial tachyarrhythmias and long-term outcomes

Outcome	Unadjusted HR (95% CI)	*p* value	Adjusted HR (95% CI)	*p* value
LR on/off AAD	2.29 (1.63–3.22)	< 0.001	2.36 (1.54–3.32)	< 0.001
LR off AAD	2.17 (1.41–3.33)	< 0.001	2.61 (1.56–4.36)	< 0.001
DCCV	2.60 (1.32–5.12)	0.006	3.63 (1.56–8.46)	0.003
Repeat ablation	3.63 (2.05–6.42)	< 0.001	4.20 (2.19–7.81)	< 0.001
Hospitalization	1.22 (0.70–2.10)	0.49	0.88 (0.44–1.77)	0.73

Abbreviations: *CI*, confidence interval; *LR*, late recurrence; *AAD*, anti-arrhythmic drug; *DCCV*, direct current cardioversion

**Fig. 2:**
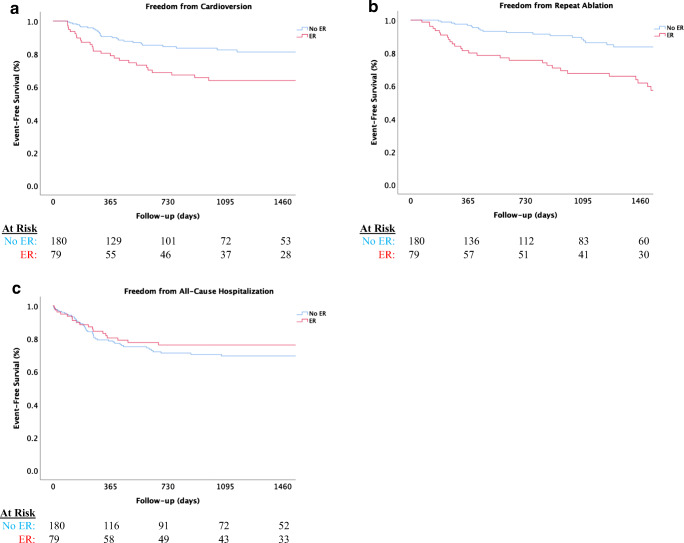
**c** Freedom from cardioversion; **d** Freedom from repeat ablation; **e** Freedom from all-cause hospitalization. Caption: Time zero indicates day of procedure.

A total of 55/259 (21.2%) patients underwent DCCV, including 23/79 (34.2%) of those with and 28/180 (15.5%) of those without ER. The mean time to DCCV of those with ER was 1560 (± 821) days and those without ER was 2077 (± 1510) days. As shown in Fig. 2a, approximately 25% of those with ER underwent cardioversion, while 17% of those without ER underwent DCCV at 4 years (log-rank *p* > 0.001). After multivariate adjustment, the risk of DCCV post-BP was higher in those with ER compared to those without ER (HR: 2.33, CI 1.19–4.56, *p* = 0.014; Table 3).

A total of 49/259 (19.1%) patients underwent repeat ablation, including 29/79 (36.7%) of those with ER and 20/180 (11.2%) of those without ER. The mean time to repeat ablation was 1549 days (± 790) in those with ER and 2196 (± 1406) days in those without ER. No patients underwent repeat ablation during the BP. Approximately 56% of those with ER and 21% of those without ER underwent repeat ablation at 4 years (log-rank *p* < 0.001), as shown in Fig. 2b. The risk of repeat ablation was fourfold higher in those with ER compared to those without ER after adjusted analysis (HR: 4.2, CI 2.19–7.81, *p* < 0.001; Table 3).

All-cause hospitalizations occurred in 79/259 (30.5%), including 18/79 (22.8%) of those with ER and 48/190 (26.7%) of those without ER. The mean time to hospitalization was 1775 (± 879) days in those with ER and 1826 (± 1086) days in those without ER with approximately 38% hospitalization rate at 4 years (log-rank *p* = 0.38), as shown in Fig. 2c. After multivariable adjustment, there was no significant difference in hospitalizations between those with ER and without ER (HR: 0.94, CI 0.55–1.76, *p* = 0.94; Table 3).

### Significance of ER timing within the blanking period

Of the 79 patients with ER, the initial timing of ER occurred in the first month in 49 patients, second month in 21 patients, and third month in 9 patients. After adjustment, the risk of LR was highest when ER occurred during the first month (HR: 2.32, CI 1.57–3.74, *p* < 0.001). A lower risk of LR was observed when ER occurred during the second month (HR: 2.01, CI 1.13–3.83, *p* = 0.019), while no signification association with LR with ER in the third month (HR: 1.46, CI 0.63–3.36, *p* = 0.374), as shown in Table 4. Despite an increased risk with LR when ER occurred earlier in the BP, ROC analysis assessing the relationship between the timing of ER and LR revealed a poor prediction (area under the curve, 0.638, *p* < 0.001). The point on the ROC curve with best discriminatory potential was 1 day (sensitivity 43.3%, specificity 83.1%).

**Table 4:** Association of early recurrence of ER and LR according to timing within the blanking period

Group	*N*	LR %	Unadjusted HR (95% CI)	*p* value	Adjusted HR (95% CI)	*p* value
No ER	177	41.8	Reference	--	Reference	--
Month 1	48	79.2	2.31 (1.56–3.43)	< 0.001	2.32 (1.57–3.74)	< 0.001
Month 2	23	65.2	2.21 (1.27–3.85)	0.005	2.01 (1.13–3.83)	0.02
Month 3	11	77.8	1.76 (0.81–2.83)	0.15	1.46 (0.63–3.36)	0.37

Abbreviations: *CI*, confidence interval; *ER*, early recurrence of atrial tachyarrhythmia; *LR*, late recurrence

## Discussion

We demonstrate several key findings in the first study examining the relationship between ER and long-term outcomes in patients following repeat ablation. First, over a median follow-up period of 3.2 years, approximately one-third of patients will experience ER following repeat ablation and the vast majority (76%) of those with ER will have LR after repeat ablation. Furthermore, less than a quarter of those with ER were free from LR at 4 years, compared to 56% of those without ER. Second, there were no predictors of ER, while ER was the strongest predictor of LR. Third, ER was strongly associated with LR, cardioversion, and repeat ablation, but not hospitalization after multivariable adjustment. Lastly, although the majority of ER occurred within the first month of the BP and was associated with over a twofold increased risk of LR, the prediction of LR based on ER timing within the BP was poor. Taken together, these findings highlight the increased risk of long-term ablation failure in those with ER and challenges clinical utilization of the 3-month BP in patients following repeat ablation.

Previous studies have demonstrated an ER incidence of approximately 38% within the 3-month BP in patients after *de novo* ablation [2]. Although approximately one-half of patients with ER may not experience LR, overwhelming data suggests that the risk of LR remains significantly higher in those with ER [2, 5, 6]. Among *de novo* ablations, the relationship persists across varying ablation strategies, including radiofrequency ablation and cryoablation, and in those with paroxysmal and persistent AF [4, 8, 11]. To the best of our knowledge, the present study is the first to examine the association between ER and LR after repeat ablation. We observed a similar incidence of ER after repeat ablation as compared to reports following *de novo* ablation; however, only less than a quarter of those with ER will not experience LR in our cohort, negating the notion that ER is benign following repeat ablation; in fact, our findings may suggest that any recurrence after repeat ablation may have clinical significance.

It has been shown that repeat ablation improves freedom from LR with approximately three-quarters of patients free of LR at 1 year with a steady decline thereafter [12, 13]. In our cohort, ER after repeat ablation was associated with over a twofold increase in LR (both on/off ADD and off AAD) over the study period. At 1 year, approximately three-quarters of those without ER were free of LR, compared to only 45% of those with ER. Various predictors of LR following repeat ablation have been previously described, such as persistent AF, hypertension, enlarged left atrium, valvular disease, and heart failure; however, the relationship with ER has not been examined [14, 15]. We found ER to be the strongest predictor of LR, along with roof line ablation. Whether the additional lesions contribute to LR or are a marker for a more advanced arrhythmogenic substrate is unclear.

Since the 3-month BP was created arbitrarily, efforts to elucidate the significance of ER within the period after *de novo* ablation have generally demonstrated that later recurrence within the BP portends to worse prognosis [5]. Willems et al. stratified 401 patients according to the last episode of ER within the BP. Those with last recurrence within the third month were associated with HR of 9.4 in predicting LR compared to a HR of 1.84 in the first month. Further ROC analysis identified 50 days as the optimal BP duration [8]. Similarly, Olshausen et al. identified 46 days as the optimal BP duration based on last episode of ER in a cohort of 713 patients who had undergone *de novo* PVI. In a Danish registry of 7339 patients, Hodges et al. demonstrated a significant graded increase of odds of LR according to the first occurrence of ER stratified by month (OR 2.08, 4.96, 5.14) [16]. While we also assessed ER according to first occurrence, we found divergent results. The majority of the patients in our cohort with ER (60%) experienced ER within the first month and were at highest risk for LR as compared to the second and third months. However, the correlation between timing of ER and LR was poor based on the ROC analysis, likely influenced by the small sample size and few event rates in the second and third months. Still, these findings suggest that patients with ER after repeat ablation typically experience ER immediately after the procedure and may be considered a failed ablation even during the BP. This may question the clinical utility of the traditional 3-month BP in patients undergoing repeat ablation.

The mechanism underlying ER following repeat ablation remains unknown. Prior studies following patients after *de novo* ablation have suggested that inflammation post-ablation, incomplete pulmonary vein isolation, non-PV triggers, or inappropriate time allotment for completion of electroanatomic changes may contribute to ER [3, 7, 17, 18]. Given the strong association between ER and LR, particularly during the first month, it argues that transient effects may have little influence on the risk relationship. In our cohort, the pulmonary veins reconnected in only approximately a quarter of patients at the time of the repeat ablation. These findings suggest that pulmonary vein isolation alone may not be sufficient to control AF in select patients as the arrhythmogenic substrate advances. Furthermore, we have shown that ER also increases the risk of cardioversion and another repeat ablation after the BP. Although studies after first-time ablation have shown that those undergoing early re-ablation strategy have improved LR freedom, further invasive rhythm control strategy for AF in patients who have already undergone repeat ablation may not always be the appropriate treatment [4, 19]. Modifiable factors such as weight loss, increasing physical activity, and reducing alcohol intake should be revisited and encouraged [20]. Additionally, further research aimed at identifying, advancing, and treating factors influencing failure of ablation, such as recognizing irreversible myopathic changes, triggers not amenable to current ablation techniques or observed with current mapping devices, or discovering unknown underlying AF mechanisms is warranted.

Our study must be interpreted in the context of several limitations inherent to its design. First, as a retrospective observational study, causal inferences cannot be made. Yet, these findings highlight a high-risk group that could benefit from further investigation. Second, the multivariable models were adjusted for available risk factors and characteristics. Other factors that may play a role in predicting outcomes, such as echocardiography characteristics, contact force sensing catheter use, and type of ER (AF vs AFL vs AT) were excluded due to missing values. Third, while all patients underwent PVI during the first ablation, data regarding adjunctive lesions from the first procedure were not captured. Finally, the type of post-ablation arrhythmia monitoring likely varied in the cohort as it was not standardized. Although this may underestimate the incidence of ER and LR, we believe that the findings remain valid as it reflects clinical practice.

## Conclusion

Following repeat AF ablation, ER is frequently observed and strongly associated with LR, cardioversion, repeat ablation, but not hospitalization. The majority of ER occurred within the first month of the BP and was associated with the highest risk of LR, although the prediction of ER timing within the BP was poor, questioning the applicability of the standard 3-month BP to AF patients following repeat ablation. Further studies are warranted to identify potential treatment strategies to reduce LR in this high-risk cohort.

## Abbreviations

AF: Atrial fibrillation
ER: Early recurrence of atrial tachyarrhythmia
PVI: Pulmonary vein isolation
LR: Late recurrence of atrial tachyarrhythmia
UCSD: University of California, San Diego
AF: Atrial flutter
AT: Atrial tachycardia
DCCV: Direct current cardioversion
ROC: Receiver operator characteristic
AAD: Antiarrhythmic drug
